# Compulsive Sexual Behaviors, Pornography Consumption, and Co-Occurring Disorders Among College Students

**DOI:** 10.1007/s10508-026-03462-w

**Published:** 2026-06-12

**Authors:** Evan J. Moon, Sheila Garos, Shane W. Kraus, Nicholas C. Borgogna

**Affiliations:** 1 Department of Psychological Sciences, Texas Tech University, Lubbock, TX 79409 USA; 2 Department of Psychology, University of Nevada, Las Vegas, Las Vegas, NV USA; 3 Department of Psychology, University of Alabama at Birmingham, Birmingham, AL USA

**Keywords:** Compulsive sexual behaviors, Depression, Alcohol use problems, Pornography consumption

## Abstract

Problematic pornography use (PPU) and other compulsive sexual behaviors (CSBs) are associated with numerous psychosocial consequences, with a higher frequency in men than women. However, less is known about the frequency and relationship between PPU, CSBs, depression, and alcohol use problems, especially among college students who may endorse these behaviors at higher rates. This study sought to identify the relationship between co-occurring CSBs, PPU, alcohol use problems, and depression across gender (77% women, 23% men) in a sample of college students (*N* = 1126). Participants completed an anonymous online survey. Categorical modeling (i.e., chi-square tests and binary logistic regressions) was utilized to identify base rates and odds ratios of clinically significant PPU and CSBs among individuals with alcohol use problems and depression. Men, relative to women, reported PPU and co-occurring alcohol use problems and depression at higher rates. Less significant gender differences were observed across co-occurring CSBs, alcohol use problems, and depression. Alcohol use problems were moderately to strongly associated with CSBs. Depression was associated with PPU in women. Co-occurring alcohol use problems and depression were moderately associated with CSBs. This study highlights the role of gender in potential comorbidities between PPU and CSBs in young adults, as well as the need for greater research examining the consequences of PPU, CSBs, and co-occurring disorders.

**Supplementary Information:**

The online version contains supplementary material available at 10.1007/s10508-026-03462-w.

## Introduction

Compulsive sexual behavior disorder (CSBD), defined as difficulties controlling intense and repetitive sexual urges and behaviors despite consequences to important areas of functioning, is a growing concern with the increased availability of the internet (Kraus et al., 2018). CSBD is associated with a multitude of negative consequences for the individual and family system. Common forms of CSBD include masturbation, cybersex, phone sex, sex with partners, pornography use, among other behaviors (Golder et al., 2023). CSBD is associated with shame (Brem et al., 2017a; Coleman et al., 2023), guilt for the sexual behaviors (Sassover et al., 2023), stigmatization (Lindsay et al., 2020), difficulties developing and sustaining romantic relationships (Starks et al., 2013), among other health problems (i.e., sexually transmitted infections; Chumakov et al., 2019; Yoon et al., 2016).

Historically, prevalence rates of CSBD have been difficult to identify, likely due to the lack of universal language, stigma, and ambiguity around the underpinnings of such behaviors (Brem et al., 2017a; Hall et al., 2025; Kürbitz & Briken, 2021). Importantly, compulsive sexual behaviors (CSBs) and CSBD are distinct, yet related constructs. CSBs are undiagnosed symptoms of CSBD, while CSBD is a formal diagnosis of behaviors and distress as defined by the diagnostic criteria of CSBD in the 11th-edition of the *International Classification of Diseases* (ICD-11; World Health Organization, 2019). Many prevalence estimates more accurately reflect compulsive sexual behav- iors or CSBD symptoms rather than the ICD-11 diagnostic criteria of CSBD, due to the use of self-report measures rather than structured diagnostic assessments. Regardless, some research suggests the prevalence rate of CSBs in adults in the USA is between 3 and 6% (Bőthe et al., 2023), while a recent study found the rate of CSBs to be as high as 10.8% (Grant et al., 2025). However, differences in prevalence estimations likely reflect differences in measurement and sampling rather than time trends (Bőthe et al., 2023).

Only recently has CSBD received recognition as an impulse-control disorder in the 11th-Edition of the International Classification of Diseases (ICD-11; World Health Organization, 2019), which has allowed for the creation of measures grounded in this diagnostic criterion (e.g., compulsive sexual behavior disorder scale; Bőthe et al., 2020). Professionally, much debate still remains over the proper classification and terminology of CSBD in diagnostic manuals (e.g., behavioral addiction or impulse-control disorder; Borgogna et al., 2022; Castro-Calvo et al., 2022; Sassover & Weinstein, 2022). Regardless of the terminology and underlying mechanisms of CSBD, empirical and theoretical evidence suggests CSBs are a growing concern and are influenced by psychological, biological, and socio-cultural factors (Coleman et al., 2018). Therefore, greater research is needed to understand the prevalence of compulsive sexual behaviors, with careful consideration for the biopsychosocial factors (e.g., gender, developmental time point) that may protect from or be risk factors for the development of CSBs.

One prominent CSBD presentation is problematic pornography use (PPU), which occurs when an individual experiences difficulties controlling intense and repetitive urges to use pornography, and results in negative consequences to their daily functioning (e.g., interferes with relationships or occupational responsibilities; Kraus et al., 2018; Wordecha et al., 2018). Among nationally representative U.S. samples, it is estimated that around 47% of men and 16% of women intentionally use pornography at least once per month (Grubbs et al., 2019), with 94.1% of men and 86.9% women reporting having viewed pornography in their lifetime (Herbenick et al., 2020). However, prevalence estimates of problematic pornography use have been much harder to identify and operationalize. The difficulty of identifying accurate estimates of PPU has been exacerbated by the lack of universal measurement approaches for identifying PPU in the general population (i.e., perceived “addiction” to pornography or differences in psychometrically validated measures; Bőthe et al., 2024; Grubbs et al., 2019). Data from the International Sex Survey which included participants from 42 countries estimated that roughly 3.2% of individuals endorse PPU (Bőthe et al., 2024). In the USA, 3.2% of individuals were above threshold on the Problematic Pornography Consumption Scale (PPCS; Bőthe et al., 2018), 10% were above threshold on the PPCS-6 (Bőthe et al., 2021), and 17% were above threshold on the Brief Pornography Screen (Bőthe et al., 2024; Kraus et al., 2020). Further, the authors note that the prevalence rates significantly differed by country, gender (i.e., men experiencing higher proportion of PPU), and measure (Bőthe et al., 2024).

### Co-Occurrence of Compulsive Sexual Behavior Disorder, and Substance Use Disorders

Research has established a link between CSBs and substance use disorders (SUDs), as a recent study found that about 24% of individuals attending in-patient treatment for SUDs reported co-occurring symptoms of CSBs (Snaychuk et al., 2024). Research is mixed regarding whether alcohol use disorder (AUD) is the most common co-occurring SUD with CSBs. Some research suggests the prevalence between co-occurring CSBs and alcohol abuse (44%) to be higher than other SUDs (22% of “other substance abuse or dependence”; Ballester-Arnal et al., 2020). Furthermore, Di Nicola et al. (2015) found that individuals with AUD were more likely to endorse at least one behavioral addiction than healthy controls. However, AUD may not be the most common co-occurring SUD in all populations, as research suggests these rates may be different across samples (i.e., clinical and college samples; Ballester-Arnal et al., 2020; Deneke et al., 2015). In a study of patients receiving intensive treatment for SUDs (i.e., in a relapse unit, primary care unit, or extended care unit), patients at risk of CSBs were significantly more likely to endorse an SUD related to marijuana (35.3% at risk of CSBs compared to 17% not at risk of CSBs), cocaine (27.5% at risk of CSBs compared to 17% not at risk), or amphetamines (23.5% at risk of CSBs compared to 8.9% not at risk of CSBs; Deneke et al., 2015).

Further research examining the base rates of co-occurring SUDs and CSBs is needed, as individuals struggling with co-occurring CSBs and SUDs may experience greater consequences and severity of symptoms for their behaviors (Efrati et al., 2024; Snaychuk et al., 2024). Di Nicola et al. (2015) found that those with an AUD and at least one behavioral addiction (e.g., CSBs or gambling), reported greater impulsivity, and more severe alcohol cravings than those without a behavioral addiction. In a Croatian sample of men (*n* = 1998), participants categorized as meeting criteria for hypersexuality had 1.8 times greater odds of experiencing substance use consequences than the non-hypersexual participants (Štulhofer et al., 2016). Research also suggests that those with a co-occurring SUD and CSBs may experience greater difficulties in attempting to navigate recovery (Snaychuk et al., 2024).

While preliminary research suggests potential links between AUD and CSBs, less research has explored a possible link between PPU and SUDs. In a sample of adolescents (*n* = 610), internet PPU was associated with sexting behaviors (i.e., engagement in sending, receiving, or posting sexually suggestive images or messages), only at high levels of alcohol consumption (Morelli et al., 2017). Although note that the sexting behaviors were not necessarily compulsive (Morelli et al., 2017). Among a sample of undergraduates (*n* = 191), significant positive correlations were identified between frequency of internet pornography use and problematic alcohol use (*r* = .19), cannabis use (*r* = .20), gambling use (*r* = .18), and video game use (*r* = .46; Harper & Hodgins, 2016). The lack of research in this area is somewhat surprising, given the association between CSBs and SUDs and the prevalence of PPU (Bőthe et al., 2024; Litam et al., 2022).

### Co-Occurrence of Compulsive Sexual Behavior Disorders, Problematic Pornography Use, Substance Use Disorders, and Depression

Another important consideration when examining the prevalence of and relationship between CSBs, PPU, and SUDs is depression. Depression may be one of the most common co-occurring disorders with CSBs and is theorized to contribute to the development and maintenance of CSBD (Ballester-Arnal et al., 2020; Engel et al., 2019b; Vieira & Griffiths, 2024; Walther et al., 2025). Many studies have revealed that those who endorse CSBs also endorse depressive symptoms at higher rates than the general population (Ballester-Arnal et al., 2020; Castro-Calvo et al., 2020; Corley & Hook, 2012; Engel et al., 2019b). For example, Ballester-Arnal et al. (2020) found that 39.7% of participants who met criteria for CSBs also met criteria for co-occurring major depressive disorder, in comparison to 22.8% who met criteria for major depressive disorder without CSBs. While it remains unclear if CSBs contribute to depressive symptoms, or if individuals who endorse depression are at a higher propensity to develop problematic sexual behaviors, research suggests the comorbidity of conditions exacerbates the problematic symptoms of CSBs, PPU, and depression (Engel, et al., 2019b; Maddock et al., 2019).

To date, little research has explored the prevalence of individuals who endorse co-occurring CSBs (i.e., PPU), SUDs, and depression comprehensively in a single analytic framework. Some research suggests individuals struggling with CSBs are more likely to experience substance use consequences and depressive symptoms (Ballester-Arnal et al., 2020; Štulhofer et al., 2016). However, findings from Deneke et al. (2015) challenge these suggestions, as individuals who were classified as “at risk” of struggling with sex addiction were significantly more likely to endorse an SUD but not a depressive disorder. Additionally, among a community sample (*n* = 113) of men, PPU was associated with depression (*r* = .29), but not alcohol problems (*r* = .08; Borgogna et al., 2025). Thus, greater research is needed to clarify these findings and to provide more up to date estimates of the frequency of CSBs and PPU in individuals who endorse SUDs and depression.

### Compulsive Sexual Behavior Disorder, Problematic Pornography Use, and Co-Occurring Disorders Across College Student Gender

College students may be an important population to examine the Co-Occurrence of CSBs, PPU, and other mental health disorders. College students are exposed to diverse and changing academic and life stressors that they are required to adapt to (Acharya et al., 2018; Pariat et al., 2014). Researchers suggest that adjustment to the demands of college may result in experimentation and the development of new coping strategies, some of which are maladaptive and may manifest in the form of substance use and pornography use (Camilleri et al., 2021; Kim & Cronley, 2020; Pariat et al., 2014). This is further supported by the high prevalence of binge drinking (Krieger et al., 2018), risky sexual behaviors (Renfro et al., 2022), and pornography use among college students (Pouralijan et al., 2024). Therefore, greater research is needed to understand the potential relationship between PPU, CSBs, alcohol use, and depressive symptoms in this population.

Across gender, research has consistently identified men as struggling with PPU and CSBs at higher rates than women (Bőthe et al., 2024; Briken et al., 2022; Dickenson et al., 2018). Additionally, some researchers have explored sex-based differences (e.g., male vs. female biological differences) while others have explored gender-based differences (i.e., social construct of how the self is viewed), which may contribute to variability in findings. Regardless, research has been limited in examining the prevalence of PPU, CSBs, and co-occurring alcohol use problems and depression across gender. Among a sample of U.S. college students (*n* = 1031), no significant differences in levels of depression or anxiety were observed across males and females who used pornography (Camilleri et al., 2021). Another study examining the relationship between pornography use and psychological outcomes in U.S. college students identified significant associations between pornography use and depression in women, and higher rates of binge drinking in men than women in the moderate to high pornography use group (Willoughby et al., 2014). Thus, it is unclear if the Co-Occurrence of CSBs, PPU, alcohol use problems, and depression will differ across men and women, and if the trend of greater PPU and CSBs in men than women will extend to these co-occurring disorders.

### Study Aims

In summary, prevalence estimates of CSBs and PPU have been hard to identify due in part to methodological and societal barriers (e.g., stigma of CSBs; Brem et al., 2017a; Hall et al., 2025; Kürbitz & Briken, 2021; Walton et al., 2017). The need for accurate estimates of CSBs and PPU in college students becomes increasingly important with the established Co-Occurrence between CSBs, substance use problems, and depression, as well as the rates of PPU, risky drinking behaviors, and depressive symptoms that have been documented in college students (Camilleri et al., 2021; Krieger et al., 2018; Sun, 2020).

The present study focuses on alcohol use problems, due to the high rate of risky drinking in college students and research that suggests it may have a particularly prominent Co-Occurrence with CSBs in non-clinical samples (Ballester-Arnal et al., 2020; Krieger et al., 2018). Furthermore, research suggests CSBs and PPU significantly differ across genders, with men reporting higher rates than women (Bőthe et al., 2024; Briken et al., 2022; Dickenson et al., 2018). However, it remains unclear if these gender differences extend to co-occurring CSBs, PPU, alcohol use problems, and depression. If these gender differences do extend to CSBs, PPU, and co-occurring disorders, this research will provide important implications for screening, assessment, and treatment of CSBs and PPU in college students. Therefore, this study attempts to expand on past research by exploring the Co-Occurrence of CSBs, PPU, alcohol use problems, and depression across men and women. The present study sought to address the following questions. Due to the exploratory nature of this study, specific hypotheses for each research question were not formulated.

#### RQ1.

 What is the frequency of PPU and CSBs in college students?

#### RQ2.

 What is the frequency of PPU and CSBs in college students who endorse alcohol use problems?

#### RQ3.

What is the frequency of PPU and CSBs in college students who endorse depression?

#### RQ4.

What is the frequency of PPU and CSBs in college students who endorse alcohol use problems and depression?

#### RQ5.

Do these above frequencies change as a function of gender?

#### RQ6.

 Are alcohol use problems associated with significantly higher CSBs and PPU?

#### RQ7.

 Is depression associated with significantly higher CSBs and PPU?

#### RQ8.

 Are co-occurring depression and alcohol use problems associated with significantly higher CSBs and PPU?

#### RQ9.

 Do these relationships change as a function of gender?

## Method

### Participants and Procedure

Participants were drawn from undergraduate subject pools at two large universities in the USA (one in the South, the other in the Mountain West). Participants self-selected to participate in a Qualtrics administered anonymous online survey assessing mental health, addiction, and sexual behaviors for small amounts of course credit. Measures were randomized to control for order effects. Initially, 1742 participants opened the survey and 1598 responses were recorded. However, we removed participants who failed custom-made random response checks used in previous studies (*n* = 339; Borgogna et al., 2023). From there, we excluded participants who did not complete at least 80% of each measure (*n* = 107), did not disclose their gender identity (*n* = 101), or were outliers on any measure (*z* > 3.29, *n* = 45 *z*-scores flagged as univariate outliers). Furthermore, we also excluded participants who held a non-cisgender identity (*n* = 17 were removed) for a few reasons, which included (1) the inability to examine relationships in a non-cisgender sample due to a lack of power, (2) the use of self-report measures leading to the potential over pathologization of LGBTQIA + individuals (Jennings et al., 2024), and (3) the differential prevalence of depression (Lu et al., 2025), alcohol use problems (Schipani-McLaughlin et al., 2022), CSBs (Bőthe et al., 2023), and PPU (Bőthe et al., 2024) between non-cisgender and cisgender individuals, potentially conflating or weakening the strength of observed relationships in the overall sample. However, see supplementary Table 1 for descriptive statistics for the main variables (i.e., CSBs, PPU, depression, and alcohol use problems) in the non-cisgender participants who were excluded from analyses. Together *n* = 1126 had data fit for analyses (*n* = 871 women, *n* = 255 men). See Table 1 for a full breakdown of participant demographics.

**Table 1:** Demographics

	Men		Women	
	*n* = 255	22.65%	*n* = 871	77.35%
*Race*				
White	184	72.16%	716	82.2%
Black or African American	24	9.41%	69	7.92%
Asian or Asian American	19	7.45%	31	3.56%
Native American or Indigenous Peoples	1	0.39%	7	.8%
Hawaiian Native or Pacific Islander	1	0.39%	4	.46%
Middle Eastern	4	1.57%	5	.57%
Multiracial	12	4.71%	25	2.87%
Self-Identify	5	1.96%	7	0.8%
Prefer Not to Specify	5	1.96%	7	0.8%
*Sexual orientation*				
Heterosexual	234	91.76%	745	85.53%
Gay/Lesbian	6	2.35%	15	1.72%
Bisexual	10	3.92%	71	8.15%
Asexual	1	0.39%	8	.92%
Pansexual	3	1.18%	10	1.15%
Prefer Not to Specify	1	.39%	7	0.8%
Questioning			11	1.26%
*Age* (in years)				
17–19	140	54.9%	630	72.33%
20–22	103	40.39%	199	22.85%
23 +	12	4.71%	40	4.59%
Missing Values			2	0.23%

The mean age was *M* = 19.47 (SD = 2.62) for the total sample, *M* = 19.70 (SD = 1.81) for men, and *M* = 19.40 (SD = 2.81) for women

### Measures

#### Brief Pornography Screen (BPS)

The 5-item BPS (Kraus et al., 2020) was used to assess PPU. The BPS is used to assess PPU within the past six months, with a score of four or higher indicating possible PPU. Participants respond to each item on a 3-point Likert scale (i.e., 0 = never, 1 = occasionally, 2 = very often). The BPS has demonstrated adequate internal consistency (Cronbach’s α = .89–.90) and validity (Kraus et al., 2020). A higher summed score on the BPS indicates greater PPU (i.e., with a maximum possible score of 10). A score of four or greater indicates probable PPU. Numerous studies have utilized similar thresholds in college student samples (Borgogna et al., 2024; Shegedin et al., 2025). The BPS has demonstrated adequate measurement invariance across males and females in prior research (Borgogna et al., 2024). The BPS demonstrated adequate internal consistency for the total sample (Cronbach’s α = .92), and across men (Cronbach’s α = .90) and women (Cronbach’s α = .89).

#### Compulsive Sexual Behavior Disorder Scale (CSBD-19)

The 19-item CSBD-19 (Bőthe et al., 2020) was used to assess clinically significant levels of CSBs. The CSBD-19 was created after the inclusion of CSBD in the ICD-11 and assesses CSBs in the past six months across five domains (i.e., control, salience, relapse, dissatisfaction, and negative consequences). Participants respond to each item on a 4-point Likert scale (i.e., 1 = totally disagree, 4 = totally agree) with higher summed scores indicating greater levels of CSBs. A score of 50 or greater suggests individuals are at high risk of struggling with clinically significant CSBs (i.e., with a maximum score of 76). The CSBD-19 was designed to be used in a wide range of populations (Bőthe et al., 2020), and other studies have used similar thresholds to detect CSBs in college student samples (Grubbs et al., 2023). The CSBD-19 has demonstrated adequate reliability (Cronbach’s α = .94) and validity in community samples, and measurement invariance across men and women (Bőthe et al., 2020). The CSBD-19 demonstrated adequate internal consistency for the total sample (Cronbach’s α = .92), and across men (Cronbach’s α = .93) and women (Cronbach’s α = .92).

#### Alcohol Use Disorder Identification Test (AUDIT)

The 10-item AUDIT (Saunders et al., 1993) was used to identify clinically significant levels of AUD. The AUDIT measures alcohol use on three domains, which include harmful alcohol use, symptoms of dependence, and hazardous alcohol use. Due in part to the elimination of alcohol dependence in the DSM-5, researchers have attempted to identify adequate cutoffs for mild, moderate, and severe AUDs. More recent research suggests a cutoff of five to seven for probable AUD (Ingesson-Hammarberg et al., 2024; Moehring et al., 2019). Ingesson-Hammarberg et al. (2024) propose a summed score of seven or higher indicates probable mild AUD in men and women, while a summed score of 16 in women and 18 in men indicates a probable moderate to severe AUD. Thus, a summed score of seven out of 40 possible points will be used as a cutoff to identify probable alcohol use problems. Numerous studies have utilized similar thresholds in college student samples (Busby et al., 2020; Kokotailo et al., 2004; Paulus et al., 2020). The AUDIT has demonstrated good internal consistency (Cronbach’s α = .75–.84) and validity (Ingesson-Hammarberg et al., 2024; Källmén et al., 2019). Furthermore, the AUDIT has demonstrated adequate measurement invariance across men and women (Skogen et al., 2019). The AUDIT demonstrated adequate internal consistency for the total sample (Cronbach’s α = .86), and across men (Cronbach’s α = .88) and women (Cronbach’s α = .85).

#### Patient Health Questionnaire (PHQ-9)

The 9-item PHQ-9 (Kroenke et al., 2001) was used to identify clinically significant depression in the past two weeks. Participants respond to each item on a 4-point scale (i.e., 0 = not at all; 3 = nearly every day), with a higher summed scale indicating more severe depression (i.e., summed score of 27 highest possible score). A score of 10 or greater suggests probable depression (Kroenke et al., 2001). Numerous studies have utilized similar thresholds in college student samples (Brown et al., 2024; Moskow et al., 2024). The PHQ-9 has demonstrated good reliability (Cronbach’s α = .86–.89) and validity across diverse samples (Kroenke et al., 2001, 2010), and adequate measurement invariance across males and females (Saunders et al., 2023). The PHQ-9 demonstrated adequate internal consistency for the total sample (Cronbach’s α = .89), and across men (Cronbach’s α = .86) and women (Cronbach’s α = .90).

#### Gender Identity

Gender identity was measured using a self-report item, “Which best represents your gender?” Response options included “Man,” “Woman,” “Transgender Man-to-Woman,” “Transgender Woman-to-Man,” “Non-binary (Male Assigned at Birth),” “Non-Binary (Female Assigned at Birth),” and “Self-Identify.” Due to the lack of gender diversity in the sample (only *n* = 17 non-cisgender individuals), concerns for the over-diagnosis of CSBs in LGBTQIA+ individuals, and the differential rates of mental health symptoms potentially impacting results, gender differences were only examined across men and women and non-cisgender individuals were excluded from the overall analyses. However, it should be noted that the exclusion of non-cisgender individuals precludes our ability to generalize these findings to non-cisgender populations.

### Data Analysis

Categorical modeling was used to identify the frequency and relationship between PPU, CSBs, alcohol use problems, and depression across men and women. PPU was categorized using the clinically significant threshold of the BPS (0 = not clinically significant, 1 = clinically significant PPU). CSB was categorized using the clinically significant threshold of the CSBD-19 (0 = not clinically significant CSB, 1 = clinically significant CSB). Alcohol use problems were categorized using the clinically significant threshold (summed score ≥ 7) of the AUDIT (0 = not clinically significant alcohol use problems, 1 = clinically significant alcohol use problems). Depression was categorized using the clinically significant threshold of the PHQ-9 (0 = not clinically significant depression, 1 = clinically significant depression). A categorical approach using thresholds to create categorical groups (i.e., not clinically significant and clinically significant) was utilized rather than a continuous approach to more adequately identify how clinical presentations may cluster.

To answer research questions 1–5, base rates were estimated to determine the frequency of clinically significant CSBs and PPU (RQ1), CSBs and co-occurring alcohol use problems as well as PPU and co-occurring alcohol use problems (RQ2), CSBs and co-occurring depression as well as PPU and co-occurring depression (RQ3), CSBs and co-occurring alcohol use problems and depression as well as PPU and co-occurring alcohol use problems and depression (RQ4) in college students, as well as across men and women (RQ5). To test research question six, we conducted two binary logistic regressions and accompanied chi-square tests with alcohol use problems as the explanatory variable in both models and CSB as the outcome variable in the first model, and PPU as the outcome variable in the second model. Similar models were constructed to test research question seven (depression as the explanatory variable and CSBs as outcome, depression as the explanatory variable and PPU as outcome), research question eight (co-occurring alcohol use problems and depression as the explanatory variable and CSBs and PPU as the outcomes), and research question nine, in which each model was split across gender.

For ease of interpretation, (*B*) coefficients were exponentiated into odds ratios (ORs) and 95% confidence intervals were used to determine statistical significance. A 95% CI that does not include one indicates statistical significance (Harris, 2021). Researchers consistently suggest ORs closer to one are not practically significant and can be described as weak, with some suggesting ORs of 1.5–2.5 are moderate, 2.5–4 are strong, and ORs > 4 are very strong (Haddock et al., 1998; Rosenthal, 1996). Some researchers have utilized and acknowledge more conservative cutoffs (i.e., *OR* < 2 not “practically significant”; Hartveit Hosøy et al., 2025; Shegedin et al., 2025). Therefore, descriptive terms were utilized to describe the strength of the ORs. These included weak (*OR* < 2), moderate (*OR* = 2–3), strong (*OR* = 3–4), and very strong (*OR* > 4; Ferguson, 2009; Rosenthal, 1996). Multicollinearity was assessed prior to analysis (i.e., highest VIF = 1.04). Z-scores ± 3.29 were considered univariate outliers and were excluded from analysis (Leys et al., 2019). The internal consistency of each scale was assessed prior to analysis (Cronbach’s *a* > .7). Lastly, participants who completed less than 80% of each measure were excluded from analysis in listwise fashion. The data was analyzed using the statistical software IBM SPSS (v.29).

## Results

The demographics of the sample are displayed in Table 1. Results of the chi-square tests and base rates for CSBs in the entire sample are displayed in Table 2. To address research questions 1–4, the following chi-square tests and base rates were used to identify the frequency and Co-Occurrence of CSBs, PPU, alcohol use problems, and depression. Of the entire sample (*n* = 1,126), 4.80% (*n* = 54) reported clinically significant CSBs, 2.13% (*n* = 24) clinically significant CSBs and depression, 3.11% (*n* = 35) clinically significant CSBs and alcohol use problems, and 1.51% (*n* = 17) reported clinically significant CSBs, alcohol use problems, and depression. For PPU in the sample, 15.45% (*n* = 174) reported clinically significant PPU, 5.60% (*n* = 63) clinically significant PPU and depression, 6.22% (*n* = 70) clinically significant PPU and alcohol use problems, and 2.93% (*n* = 33) reported clinically significant PPU, alcohol use problems, and depression (see Table 3). See supplementary Table 2 for base rates by gender of depression, alcohol use problems, and co-occurring depression and alcohol use problems among individuals who endorsed PPU and CSBs. See supplementary Table 3 for base rates by gender of CSBs and PPU among individuals who endorsed depression, alcohol use problems, and co-occurring depression and alcohol use problems.

**Table 2:** Chi-square and base rates of compulsive sexual behaviors in students

	No CSBs [*n* (%)]	CSBs [*n* (%)]	χ^2^ (df)	*p*	Φ
PHQ-9			2.23 (1)	.136	0.04
At or above clinical threshold	370 (34.51%)	24 (44.44%)			
Below clinical threshold	702 (65.49%)	30 (55.55%)			
AUDIT-10			23.47 (1)	< .001	0.14
At or above clinical threshold	351 (32.74%)	35 (64.81%)			
Below clinical threshold	721 (67.26%)	19 (35.19%)			
AUDIT-10 + PHQ-9			11.88 (1)	< .001	0.10
At or above clinical threshold	153 (14.27%)	17 (31.48%)			
Below clinical threshold	919 (85.73%)	37 (68.52%)			

PHQ-9: Patient Health Questionnaire-9, AUDIT-10: Alcohol Use Disorder Identification Test-10, CSBs assessed using the Compulsive Sexual Behavior Disorder Scale-19

**Table 3:** Chi-square and base rates of problematic pornography use in students

	No PPU [*n* (%)]	PPU [*n* (%)]	χ^2^ (df)	*p*	Φ
PHQ-9			.134 (1)	.715	0.01
At or above clinical threshold	331 (34.77%)	63 (36.21%)			
Below clinical threshold	621 (65.23%)	111 (63.79%)			
AUDIT-10			3.23 (1)	.072	0.05
At or above clinical threshold	316 (33.19%)	70 (40.23%)			
Below clinical threshold	636 (66.81%)	104 (59.77%)			
AUDIT-10 + PHQ-9			2.40 (1)	.121	0.05
At or above clinical threshold	137 (14.39%)	33 (18.97%)			
Below clinical threshold	815 (85.61%)	141 (81.03%)			

PHQ-9: Patient Health Questionnaire-9, AUDIT-10: Alcohol Use Disorder Identification Test-10, problematic pornography use assessed using the Brief Pornography Screen

In line with research question five, the following chi-square tests and base rates aimed to identify if these frequencies differed across gender. Results of the chi-square tests and base rates across gender are displayed in Table 4. For CSBs among men (*n* = 255), 7.84% (*n* = 20) reported clinically significant CSBs, 2.35% (*n* = 6) clinically significant CSBs and depression, 5.10% (*n* = 13) clinically significant CSBs and alcohol use problems, and 1.57% (*n* = 4) reported clinically significant CSBs, alcohol use problems, and depression. Of the men who reported CSBs (*n* = 20), 30.00% reported depression, 65.00% reported alcohol use problems, and 20.00% reported co-occurring depression and alcohol use problems. For PPU among men, (*n* = 255), 45.49% (*n* = 116) reported clinically significant PPU, 11.76% (*n* = 30) clinically significant PPU and depression, 20.00% (*n* = 51) clinically significant PPU and alcohol use problems, and 7.45% (*n* = 19) reported clinically significant PPU, alcohol use problems, and depression. Of the men who reported PPU (*n* = 116), 25.86% reported depression, 43.97% reported alcohol use problems, and 16.38% reported co-occurring depression and alcohol use problems.

**Table 4:** Chi-square statistic and base rates by gender

	Men [*n* (%)]	Women [*n* (%)]	χ^2^ (df)	*p*	Φ
CSBD-19			6.71 (1)	.010	− 0.08
At or above clinical threshold	20 (7.84%)	34 (3.90%)			
Below clinical threshold	235 (92.16%)	837 (96.10%)			
BPS			227.65 (1)	< .001	− 0.45
At or above clinical threshold	116 (45.49%)	58 (6.66%)			
Below clinical threshold	139 (54.51%)	813 (93.34%)			
CSBD-19 + AUDIT-10			4.33 (1)	.037	− 0.06
At or above clinical threshold	13 (5.10%)	22 (2.53%)			
Below clinical threshold	242 (94.90%)	849 (97.47%)			
BPS + AUDIT-10			107.42 (1)	< .001	− 0.31
At or above clinical threshold	51 (20%)	19 (2.18%)			
Below clinical threshold	204 (80%)	852 (97.82%)			
CSBD-19 + PHQ-9			0.08 (1)	.781	− 0.01
At or above clinical threshold	6 (2.35%)	18 (2.07%)			
Below clinical threshold	249 (97.65%)	853 (97.93%)			
BPS + PHQ-9			23.76 (1)	< .001	− .15
At or above clinical threshold	30 (11.76%)	33 (3.79%)			
Below clinical threshold	225 (88.24%)	838 (96.21%)			
CSBD-19 + AUDIT-10 + PHQ-9			0.01 (1)	.930	− .003
At or above clinical threshold	4 (1.57%)	13 (1.49%)			
Below clinical threshold	251 (98.43%)	858 (98.51%)			
BPS + AUDIT-10 + PHQ-9			23.68 (1)	< .001	− .15
At or above clinical threshold	19 (7.45%)	14 (1.61%)			
Below clinical threshold	236 (92.55%)	857 (98.39%)			

CSBD-19: Compulsive Sexual Behavior Disorder Scale-19, BPS: Brief Pornography Screen, PHQ-9: Patient Health Questionnaire-9, AUDIT-10: Alcohol Use Disorder Identification Test-10

For CSBs among women (*n* = 871), 3.90% (*n* = 34) reported clinically significant CSBs, 2.10% (*n* = 18) clinically significant CSBs and depression, 2.53% (*n* = 22) clinically significant CSBs and alcohol use problems, and 1.49% (*n* = 13) reported clinically significant CSBs, alcohol use problems, and depression. Of the women who reported CSBs (*n* = 34), 52.94% reported depression, 64.71% alcohol use problems, and 38.24% co-occurring depression and alcohol use problems. For PPU among women (*n* = 871), 6.66% (*n* = 58) reported clinically significant PPU, 3.79% (*n* = 33) clinically significant PPU and depression, 2.18% (*n* = 19) clinically significant PPU and alcohol use problems, and 1.61% (*n* = 14) reported clinically significant PPU, alcohol use problems, and depression. Of the women who reported PPU (*n* = 58), 56.90% reported depression, 32.75% alcohol use problems, and 24.14% co-occurring depression and alcohol use problems.

The following binary logistic regressions aimed to address research questions 6–8, and examined if clinically significant depression, alcohol use problems, and co-occurring depression and alcohol use problems were associated with higher odds of CSBs and PPU. For the overall sample, clinically significant alcohol use problems were strongly associated with increased odds of reporting clinically significant CSBs and weakly associated with an increase in the odds of reporting clinically significant PPU. Clinically significant depression was weakly associated with an increase in the odds of clinically significant CSBs and weakly associated with an increase in the odds of clinically significant PPU. Clinically significant alcohol use problems and depression were moderately associated with an increase in the odds of clinically significant CSBs and weakly associated with an increase in the odds of clinically significant PPU. See Table 5 for results of the binary logistic regressions in the overall sample.

**Table 5:** Results of binary logistic regression across students

	CSBs					PPU				
Predictor	*OR*	95% CI	*p*_1_	χ^2^ (df)	*p*_2_	*OR*	95% CI	*p*_3_	χ^2^ (df)	*p*_4_
Above Threshold on AUDIT-10	3.78	2.13–6.71	< .001	21.98 (1)	< .001	1.36	0.97–1.89	.073	3.17 (1)	.075
Above Threshold on PHQ-9	1.52	0.88–2.63	.138	2.16 (1)	.142	1.07	0.76–1.49	.715	0.13 (1)	.715
Above Threshold on AUDIT-10 and PHQ-9	2.76	1.52–5.03	< .001	9.70 (1)	.002	1.39	0.92–2.12	.122	2.28 (1)	.131

PHQ-9: Patient Health Questionnaire-9, AUDIT-10: Alcohol Use Disorder Identification Test-10, CSBs assessed using Compulsive Sexual Behavior Disorder Scale-19, PPU assessed using the Brief Pornography Screen. *OR* = Odds Ratio, χ^2^ = Chi-Square value for overall model, *p*_1_ = *p*-value for each *OR* in CSBs*, p*_2_ = *p*-value for each χ^2^ in CSBs, *p*_3_ = *p*-value for each *OR* in PPU, *p*_4_ = p-value for each χ^2^ in PPU

The following binary logistic regressions were utilized to examine research question eight to identify if the relationships between alcohol use problems, depression, CSBs, and PPU differ across gender. See Table 6 for results of the binary logistic regressions for CSBs across gender. See Table 7 for results of the binary logistic regressions for PPU across gender. For men, clinically significant alcohol use problems were moderately associated with an increase in the odds of clinically significant CSBs. Clinically significant depression was weakly associated with an increase in the odds of clinically significant PPU. Clinically significant alcohol use problems and depression were weakly associated with an increase in the odds of clinically significant CSBs, although this relationship should be interpreted cautiously due to insufficient separation across conditions (i.e., *n* = 4 in cell with CSBs, alcohol use problems, and depression). Clinically significant alcohol use problems and depression were weakly associated with an increase in the odds of clinically significant PPU.

**Table 6:** Results of binary logistic regression for compulsive sexual behaviors across gender

	CSBs (Men)					CSBs (Women)					
Predictor	*OR*	95% CI	*p*_1_	χ^2^ (df)	*p*_2_	*OR*		95% CI	*p*_3_	χ^2^ (df)	*p*_4_
Above Threshold on AUDIT-10	2.69	1.04–6.99	.042	4.35 (1)	.037	4.18		2.04–8.58	< .001	16.14 (1)	< .001
Above Threshold on PHQ-9	1.47	.54–4.02	.451	0.54 (1)	.461	1.85		0.93–3.67	.081	3.04 (1)	.081
Above Threshold on AUDIT-10 and PHQ-9	1.59	.50–5.05	.435	0.57 (1)	.452	3.66		1.79–7.51	< .001	11.01 (1)	< .001

PHQ-9: Patient Health Questionnaire-9, AUDIT-10: Alcohol Use Disorder Identification Test-10, CSBs assessed using Compulsive Sexual Behavior Disorder Scale-19. *OR* = Odds Ratio, χ^2^ = chi-Square value for overall model, *p*_1_ = *p*-value for each *OR* in men*, p*_2_ = *p*-value for each χ^2^ in men, *p*_3_ = *p*-value for each *OR* in women, *p*_4_ = *p*-value for each χ^2^ in women

**Table 7:** Results of binary logistic regression for problematic pornography use across gender

	PPU (Men)					PPU (Women)				
Predictor	*OR*	95% CI	*p*_1_	χ^2^ (df)	*p*_2_	*OR*	95% CI	*p*_3_	χ^2^ (df)	*p*_4_
Above Threshold on AUDIT-10	1.10	0.67–1.80	.719	0.13 (1)	.719	1.05	0.59–1.85	.871	.03 (1)	.872
Above Threshold on PHQ-9	1.32	0.74–2.37	.347	0.89 (1)	.347	2.23	1.30–3.83	.003	8.64 (1)	.003
Above Threshold on AUDIT-10 and PHQ-9	1.41	0.69–2.85	.345	0.89 (1)	.344	1.84	0.98–3.46	.059	3.25 (1)	.071

PHQ-9: Patient Health Questionnaire-9, AUDIT-10: Alcohol Use Disorder Identification Test-10, problematic pornography use assessed using the Brief Pornography Screen. *OR* = Odds Ratio, χ^2^ = chi-Square value for overall model, *p*_1_ = *p*-value for each *OR* in men*, p*_2_ = *p*-value for each χ^2^ in men, *p*_3_ = *p*-value for each *OR* in women, *p*_4_ = *p*-value for each χ^2^ in women

For women, clinically significant alcohol use problems were very strongly associated with an increase in the odds of clinically significant CSBs and weakly associated with an increase in the odds of clinically significant PPU. Clinically significant depression was weakly associated with an increase in the odds of clinically significant CSBs and moderately associated with an increase in the odds of clinically significant PPU. Clinically significant alcohol use problems and depression were strongly associated with an increase in the odds of clinically significant CSBs and weakly associated with an increase in the odds of clinically significant PPU.

## Discussion

In the present study, we examined the frequency and relationship between CSBs, PPU, alcohol use problems, and depression among college students. Consistent with past research, 4.80% of individuals endorsed CSBs and 15.45% endorsed PPU (Bőthe et al., 2020, 2024; Odlaug et al., 2013). Results of the chi-square tests suggest gender may have a unique relationship with PPU, alcohol use problems, and depression. In comparison to women, men endorsed PPU (45.49% vs. 6.66%), PPU and alcohol use problems (20.00% vs. 2.18%), PPU and depression (11.76% vs. 3.79%), and co-occurring PPU, alcohol use problems, and depression (7.45% vs. 1.61%) at consistently higher rates. Although research has primarily examined CSBs within the context of these disorders over and above PPU, aspects of these findings are consistent with past research, as men have been found to endorse PPU and alcohol use problems at higher rates than women (Bőthe et al., 2024; Foster et al., 2015).

In line with researchers who have suggested that PPU may manifest as a maladaptive coping strategy, this gender disparity could suggest that young adult college men may be more likely to turn to pornography to cope with the stressors of adjusting to life in college than women (Testa et al., 2024). However, note that the percentage of men who endorsed PPU is significantly higher than previous research (Bőthe et al., 2024), which could be a result of factors such as moral incongruence (Grubbs & Floyd, 2025), religiosity, or the use of the Brief Pornography Screen using a relatively mild detection threshold (i.e., a less comprehensive measure of PPU; Kraus et al., 2020). Additionally, the frequency of co-occurring depression, as well as co-occurring depression and alcohol use problems in individuals who endorsed PPU and CSB was consistently more frequent in women than men. For example, approximately 56.90% of women and 25.86% of men who endorsed PPU also endorsed co-occurring depression. In fact, only co-occurring alcohol use problems were more frequent in men who endorsed PPU (43.97%) and CSBs (65.00%) than women who endorsed PPU (32.76%) and CSBs (64.71%). These findings suggest that while PPU, CSBs, and co-occurring psychopathology may be more widespread in men than women likely due to higher rates of PPU and CSBs in men, co-occurring psychopathology may be more concentrated in women presenting with PPU and CSBs than men. This finding could suggest that PPU and CSBs are more distressing in women than men, potentially due to social stigma, decreased help-seeking attitudes for PPU and CSBs, or gender specific underlying transdiagnostic mechanisms (Fraumeni-McBride, 2023). However, these results should be interpreted with caution, as diagnostic data using structured clinical assessments would likely lead to more conservative estimates of PPU.

Further, results of the binary logistic regressions suggest alcohol use problems, depression, and their Co-Occurrence are weakly associated with increased odds of PPU among men, while clinically significant depression among women was moderately associated with increased odds of PPU. This finding is interesting, as depression displayed weak associations with PPU in the overall sample and among men. This could suggest that depression is uniquely associated with PPU in women, which is supported by previous research (Willoughby et al., 2014). Additionally, these results suggest women presenting with depression may benefit from assessments of PPU. These results also provide evidence for a relationship between PPU and depression, but not alcohol use problems. The lack of relationship between PPU and alcohol use problems across the overall sample, men, and women is surprising given PPU is a manifestation of CSBs (Ballester-Arnal et al., 2020). Possible explanations for the lack of relationship between alcohol use problems and PPU could be that alcohol use actually decreases symptoms of PPU (e.g., frequency, amount, distress) because (1) attention is spread across both alcohol use and pornography use, (2) the underlying motivation for the behavior (i.e., emotion regulation, coping, pleasure) is being satisfied by the use of alcohol and pornography, (3) alcohol use problems may be more salient for individuals, leading to less distress regarding their pornography use and the perception that their use is not “problematic,” and (4) individuals with greater alcohol use may experience less moral incongruence and moral distress for their pornography use. However, the explanations offered for the lack of relationship between alcohol use problems and PPU are speculative, and researchers should explore the underlying mechanisms in future research.

Some research supports the unique association between PPU and depression among women, as women who engage in pornography have been found to experience greater distress, and stronger associations with depression (Tan et al., 2022; Willoughby et al., 2014). Gendered coping strategies, social norms, and stigma could explain the unique relationship between depression and PPU in women (Katz-Wise & Hyde, 2014; Koós et al., 2025). For example, societal norms and pressures that portray PPU and CSBs as a predominantly “male” struggle could contribute to greater psychological distress among women struggling with PPU (Katz-Wise & Hyde, 2014). Inversely, this finding could also suggest that women struggling with depression may be more likely to turn to PPU as a coping strategy than men. Nevertheless, greater research is needed to understand why this association is stronger in women than men and future research should consider examining societal messages and motivations for pornography use across men and women struggling with depression.

Less significant gender differences were observed between CSBs, alcohol use problems, and depression. In comparison to women, men endorsed CSBs (7.84% vs. 3.90%) and co-occurring CSBs and alcohol use problems (5.10% vs. 2.53%) at higher rates. This finding is consistent with past research, as clinically significant CSBs (i.e., using the CSBD-19) have been reported at lower rates than PPU (i.e., using the BPS), and men typically endorse CSBD at higher rates than women (Wizła et al., 2022). This lower frequency of CSBs and associated co-occurring disorders in comparison to PPU is likely a result of the measure, as the BPS (Kraus et al., 2020) is a brief screening measure of PPU, while the CSBD-19 is a more extensive measure of CSBs (Bőthe et al., 2020, 2024).

Additionally, alcohol use problems were moderately associated with increased odds of CSBs in men, and very strongly associated with increased odds of CSBs in women. Co-occurring alcohol use problems and depression were strongly associated with increased odds of CSBs in women. Similar findings were reported in the overall sample, as alcohol use problems were strongly associated with CSBs, and co-occurring alcohol use problems and depression were moderately associated with CSBs. The finding that alcohol use problems were associated with CSBs more strongly in women than men likely reflects (1) the closing gender disparity in rates of alcohol use problems between young adult men and women, as well as (2) the increased rates of psychiatric comorbidity (e.g., SUDs, panic disorder) and consequences women with alcohol use problems may experience relative to men (Fonseca et al., 2021; Lespine et al., 2022; White, 2020). This research further supports the established relationship between co-occurring CSBs and alcohol use problems, as well as provides novel contributions to suggest a relationship between co-occurring CSBs, alcohol use problems, and depression exists and should be explored in greater depth in future research (Ballester-Arnal et al., 2020; Štulhofer et al., 2016; Sussman et al., 2011). Potential explanations for the relationship between CSBs and alcohol use problems could be underlying transdiagnostic mechanisms, such as experiential avoidance (Brem et al., 2018), impulsivity (Levi et al., 2020; Stamates et al., 2022), and emotion dysregulation (Lew-Starowicz et al., 2020). These findings suggest mental health professionals should consider utilizing assessments for CSBs in young adult men and women presenting with alcohol use problems (i.e., AUD), and women presenting with co-occurring alcohol use problems and depression.

Unfortunately, little research has been devoted to identifying the transdiagnostic mechanisms that contribute to the development of these co-occurring disorders, as well as the consequences of struggling with co-occurring alcohol use problems, depression, and CSBs. This is concerning, given that researchers have suggested that co-occurring disorders with CSBs may increase the severity of CSB related consequences (Engel, et al., 2019b; Maddock et al., 2019). Researchers should consider utilizing a dimensional approach when exploring the Co-Occurrence of these disorders to further understand the transdiagnostic mechanisms (i.e., shared and additive pathways) that may contribute to the development, maintenance, and exacerbated symptomology of CSBs and PPU with co-occurring psychopathology. For example, some researchers have suggested that this link could be explained by constructs such as experiential avoidance, which have been found to mediate the relationship between psychopathology and CSBs in individuals struggling with substance use (Brem et al., 2017b, 2018). Greater understanding of the transdiagnostic mechanisms (e.g., experiential avoidance) between CSBs, PPU, and co-occurring disorders could increase the efficacy of assessment detection, treatment, interventions, and prevention methods.

These results have particular relevance for clinicians in university settings, as these findings suggest the relationship between CSBs, PPU, alcohol use problems, and depression is different across men and women. These results suggest university clinicians should assess for depression in young adult women presenting with PPU and CSBs, as well as alcohol use problems in young adult men and women presenting with CSBs. Assessing for these co-occurring disorders while considering the unique aspects of identity, social norms, and stigma that influence the presentation of symptoms will likely lead to more effective treatment. Given the frequency of PPU, CSBs, and co-occurring alcohol use problems in our sample, college campuses may also consider increasing student accessibility to on-campus support groups for students struggling with PPU, CSBs, and alcohol use problems. Additionally, the higher-than-average rates of PPU among young adult men in the present study suggests that university clinicians should consider assessing for factors such as moral incongruence, religiosity, and sexual orientation, which have been found to lead to the over pathologization of sexual behaviors (e.g., CSBs; Droubay et al., 2025; Grubbs & Floyd, 2025). Lastly, university clinicians should consider assessing for transdiagnostic processes (e.g., experiential avoidance and emotion dysregulation) that may contribute to the Co-Occurrence of PPU, CSBs, alcohol, and depression (Brem et al., 2017b, 2018).

### Limitations

The present study has a few important limitations. The first limitation includes the lack of gender diversity in the sample, which limited our ability to examine the frequency of these disorders in gender diverse individuals. Future research should address this limitation, as PPU and CSB research has predominantly been limited to cisgender men and women (Borgogna et al., 2022; Jennings et al., 2022). Additionally, this study established a relationship between these disorders, however, the cross-sectional design of the study inhibited the researchers ability to identify the directionality and consequences of clinically significant co-occurring disorders. Researchers should consider expanding on this study by exploring if individuals struggling with CSBs or PPU and co-occurring disorders experience decreased psychological, relational, and physical well-being than individuals solely struggling with CSBs or PPU. Relatedly, this study was unable to examine mechanistic pathways that could explain the observed relationships and potential gender differences observed in this study. Future studies should employ multi-site representativeness and pre-registration and explore the role of additive and distinct mechanistic pathways to validate and explain these observed relationships and gender differences.

Further, the present study utilized a sample of U.S. college students. Therefore, these results should be interpreted with caution when considering these findings within the context of other populations, as college students may endorse these disorders at higher rates than the general population (Camilleri et al., 2021; Sun, 2020). The use of undergraduate psychology subject pools may also have introduced selection bias and social desirability bias into the sample due to the sensitive nature of the measures and differences in students’ openness to sexuality, substance use, and psychological distress. The use of self-report data (i.e., no clinical assessment of symptoms) may also have contributed to overreporting and underreporting of participants pornography use, alcohol use, and psychological distress, which may have impacted the analyses and interpretation of the data. Additionally, the use of a “mild” cutoff for alcohol use problems on the AUDIT could have contributed to an overestimation of alcohol use problems in the sample (Ingesson-Hammarberg et al., 2024). Therefore, these findings may not be generalizable to individuals with more significant alcohol use problems, and researchers should explore how these relationships may differ at more severe levels of alcohol use problems (e.g., moderate to severe threshold on the AUDIT). Furthermore, the researchers did not account for differences across institutions that the data was collected from, which may have impacted findings.

Researchers should examine the frequencies and relationships between CSBs, PPU, alcohol use problems, and depression in other populations (e.g., adolescence) using different measures (i.e., structured assessments, behavioral measures) to identify how these relationships may differ. Doing so would have important implications for screening, increasing treatment efficacy, and the expansion of CSBD, PPU, and comorbidity research. Additionally, although a relationship was identified between the disorders in this study, the researchers were unable to draw causal conclusions due to the cross-sectional nature of this design. Future research should utilize alternative designs (i.e., qualitative and longitudinal) to identify how these disorders may contribute to the development and continuation of each other. Future research should also consider utilizing clinical assessments to diagnose and more thoroughly assess individuals for CSBD, AUD, and depression.

### Conclusions

The Co-Occurrence of PPU, alcohol use problems, and depression appears to be higher in young adult men than women. Less significant gender differences were observed across co-occurring CSBs, alcohol use problems, and depression. Meaningful associations were identified between co-occurring alcohol use problems and depression and increased odds of CSBs. Alcohol use problems displayed moderate to strong associations between CSBs in the overall sample, and across men and women. Meaningful associations between depression and PPU were only observed in women. These findings suggest the frequency and relationship between these disorders differs across gender. Further, depression may uniquely be associated with PPU in women, and alcohol use problems may be associated with CSBs but not PPU. Clinicians should consider how an individual’s identity (i.e., gender identity) and presenting concerns (i.e., alcohol use or mood) may influence decisions to assess for and treat CSBD and PPU. Greater research utilizing qualitative and longitudinal designs is necessary to understand the potential consequences and symptoms of PPU, CSBs, and co-occurring depression and alcohol use problems (i.e., AUD) in order to enhance the detection and treatment of CSBs and PPU. Additionally, qualitative and longitudinal designs would assist in understanding the lived experiences and temporal ordering of individuals who endorse co-occurring CSBs, PPU, substance use problems, and depression.

## Supplementary Information

Below is the link to the electronic supplementary material.

Supplementary file1 (DOCX 36 kb)

Supplementary file2 (DOCX 16 kb)

Supplementary file3 (DOCX 16 kb)

## Data Availability

This data are available upon request.

## Code Availability

The code and output associated with this data is available upon request.
