# Supplementary material for: Compulsive Sexual Behaviors, Pornography Consumption, and Co-Occurring Disorders Among College Students
Source: Arch Sex Behav. 2026 Jun 12;55(5):2299–315. doi: 10.1007/s10508-026-03462-w (PMC13427987; doi:10.1007/s10508-026-03462-w)
Supplement: Supplementary file 1 — Supplementary file1 (DOCX 36 kb) [file 10508_2026_3462_MOESM1_ESM.docx]

Supplementary Table 1

*Descriptive Statistics and Base Rates in Non-cisgender Individuals*

|  | [*n* (%)] | | | *M* | *SD* | |  |
| --- | --- | --- | --- | --- | --- | --- | --- |
| CSBD-19 | |  | 30.00 | | 10.88 |  |  |
| At or above clinical threshold | | 0 (0.00%) |  | |  |  |  |
| Below clinical threshold | | 17 (100.00%) |  | |  |  |  |
| BPS | |  | 1.12 | | 2.15 |  |  |
| At or above clinical threshold | | 3 (17.65%) |  | |  |  |  |
| Below clinical threshold | | 14 (82.35%) |  | |  |  |  |
| AUDIT-10 | |  | 3.47 | | 4.29 |  |  |
| At or above clinical threshold | | 3 (17.65%) |  | |  |  |  |
| Below clinical threshold | | 14 (82.35%) |  | |  |  |  |
| PHQ-9 | |  | 13.24 | | 6.69 |  |  |
| At or above clinical threshold | | 14 (82.35%) |  | |  |  |  |
| Below clinical threshold | | 3 (17.65%%) |  | |  | | |

*Note.* CSBD-19: Compulsive Sexual Behavior Disorder Scale-19, BPS: Brief Pornography Screen, PHQ-9: Patient Health Questionnaire-9, AUDIT-10: Alcohol Use Disorder Identification Test-10.
