# Supplementary material for: Compulsive Sexual Behaviors, Pornography Consumption, and Co-Occurring Disorders Among College Students
Source: Arch Sex Behav. 2026 Jun 12;55(5):2299–315. doi: 10.1007/s10508-026-03462-w (PMC13427987; doi:10.1007/s10508-026-03462-w)
Supplement: Supplementary file 2 — Supplementary file2 (DOCX 16 kb) [file 10508_2026_3462_MOESM2_ESM.docx]

Supplementary Table 2

*Base Rates By Gender of Participants Who Endorsed PPU and CSB*

|  | PPU | | CSB | |
| --- | --- | --- | --- | --- |
|  | Men [*n* (%)] | Women [*n* (%)] | Men [*n* (%)] | Women [*n* (%)] |
| PHQ-9 |  |  |  |  |
| At or above clinical threshold | 30 (25.86%) | 33 (56.90%) | 6 (30.00%) | 18 (52.94%) |
| Below clinical threshold | 86 (74.14%) | 25 (43.10%) | 14 (70.00%) | 16 (47.06%) |
| AUDIT-10 |  |  |  |  |
| At or above clinical threshold | 51 (43.97%) | 19 (32.76%) | 13 (65.00%) | 22 (64.71%) |
| Below clinical threshold | 65 (56.03%) | 39 (67.24%) | 7 (35.00%) | 12 (35.29%) |
| PHQ-9 + AUDIT-10 |  |  |  |  |
| At or above clinical threshold | 19 (16.38%) | 14 (24.14%) | 4 (20.00%) | 13 (38.24%) |
| Below clinical threshold | 97 (83.62%) | 44 (75.86%) | 16 (80.00%) | 21 (61.76%) |

*Note.* This table depicts the base rates of depression, alcohol use problems, and co-occurring depression and alcohol use problems in men and women who endorsed PPU and CSB. PPU: Brief Pornography Screener, CSB: Compulsive Sexual Behavior Disorder Scale-19, PHQ-9: Patient Health Questionnaire-9, AUDIT-10: Alcohol Use Disorder Identification Test-10.
