# Supplementary material for: Compulsive Sexual Behaviors, Pornography Consumption, and Co-Occurring Disorders Among College Students
Source: Arch Sex Behav. 2026 Jun 12;55(5):2299–315. doi: 10.1007/s10508-026-03462-w (PMC13427987; doi:10.1007/s10508-026-03462-w)
Supplement: Supplementary file 3 — Supplementary file3 (DOCX 16 kb) [file 10508_2026_3462_MOESM3_ESM.docx]

Supplementary Table 3

|  | PHQ-9 | | | | | | AUDIT-10 | | | | | | | PHQ-9 + AUDIT-10 | | | | | | |
| --- | --- | --- | --- | --- | --- | --- | --- | --- | --- | --- | --- | --- | --- | --- | --- | --- | --- | --- | --- | --- |
|  | Men [*n* (%)] | | Women [*n* (%)] | | Men [*n* (%)] | | | | Women [*n* (%)] | | | | Men [*n* (%)] | | | | Women [*n* (%)] | | |  |
| BPS |  |  | | | |  | | | |  | |  | | | |  | |  |  |  |
| At or above clinical threshold | 30 (50.85%) | 33 (9.85%) | | 51 (46.79%) | | | | 19 (6.86%) | | | 19 (52.78%) | | | | 14 (10.45%) | | | |  |  |
| Below clinical threshold | 29 (49.15%) | 302 (90.15%) | | 58 (53.21%) | | | | 258 (93.14%) | | | 17 (47.22%) | | | | 120 (89.55%) | | | |  |  |
| CSBD-19 |  |  | |  | | | |  | | |  | | | |  | | | |  |  |
| At or above clinical threshold | 6 (10.17%) | 18 (5.37%) | | 13 (11.93%) | | | | 22 (7.94%) | | | 4 (11.11%) | | | | 13 (9.70%) | | | |  |  |
| Below clinical threshold | 53 (89.83%) | 317 (94.63%) | | 96 (88.07%) | | | | 255 (92.06%) | | | 32 (88.89%) | | | | 121 (90.30%) | | | |  |  |

*Base Rates By Gender of Participants Who Endorsed Depression and Alcohol Use Problems*

*Note.* This table depicts the base rates of PPU and CSB in men and women who endorsed depression and alcohol use problems. BPS: Brief Pornography Screener, CSBD-19: Compulsive Sexual Behavior Disorder Scale-19, Depression: Patient Health Questionnaire-9, Alcohol Use Problems: Alcohol Use Disorder Identification Test-10.
